# Evaluation of risk factors for mortality in hematological malignancy patients admitted to intensive care unit

**DOI:** 10.1186/2197-425X-3-S1-A249

**Published:** 2015-10-01

**Authors:** I Ileri, R Coskun, K Gundogan, M Guven, M Sungur

**Affiliations:** Internal Medicine, Erciyes University Faculty of Medicine, Kayseri, Turkey; Internal Medicine Intensive Care Unit, Erciyes University Faculty of Medicine, Kayseri, Turkey

## Intr

Haematological malignancies are associated with treatment releated morbidity and mortality. Intensive chemotherapy and haematopoetic stem cell transplantation has increased treatment releated complications. Transferring a severely ill patients to the intensive care unit for life support is often a difficult decision.

## Objectives

To detect risk factors effecting haematological malignancy patients mortality after admission to the medical intensive care unit.

## Methods

This study was performed prospectively in the medical intensive care unit of Erciyes Universty Hospital. History, physical exam and laboratory findings on admisssion,and therapeutic interventions during ICU stay were recorded. The study end point was ICU mortality.

## Results

32 (%60) of the 53 patients included into this study were male. The average age of the patients was 49 ± 19 years. The hematological diagnosis of the patients were as follows; 19 were AML, 12 were MM, 7 were Non-Hodgin Lymphoma and 6were ALL. The most common reasons for ICU admissions were respiratory failure (%57) and septic shock (%17). The mean time delay starting from deteriation to ICU was 6 hours (range 1-48). APACHE-2 score was 26 ± 8 and the early warning system score was 8 (range 1-14). APACHE-2 score and time delay for ICU admission of nonsurvivors were higher (p= 0.009, 0.05 respectively). Serum total cortisol levels were lower in survivors compared to nonsurvivors (p= 0.023). ICU mortality rate was 60%.

## Conclusions

The mortality rate of haematological malignancy patients rate was high. The time delay for ICU admission and APACHE-2 score were important risk factors for ICU mortality. These patients should be admitted to the ICU as soon as possible when vital signs are detoriated.

